# Prognostic Impact of Plasma Epstein-Barr Virus DNA in Patients with Nasopharyngeal Carcinoma Treated using Intensity-Modulated Radiation Therapy

**DOI:** 10.1038/srep22000

**Published:** 2016-02-29

**Authors:** Hao Peng, Rui Guo, Lei Chen, Yuan Zhang, Wen-Fei Li, Yan-Ping Mao, Ying Sun, Fan Zhang, Li-Zhi Liu, Ai-Hua Lin, Jun Ma

**Affiliations:** 1 Department of Radiation Oncology, Sun Yat-sen University Cancer Center, State Key Laboratory of Oncology in Southern China, Collaborative Innovation Center for Cancer Medicine, People’s Republic of China; 2 Imaging Diagnosis and Interventional Center, Sun Yat-sen University Cancer Center, State Key Laboratory of Oncology in Southern China, Collaborative Innovation Center for Cancer Medicine, People’s Republic of China; 3 Department of Medical Statistics and Epidemiology, School of Public Health, Sun Yat-sen University, People’s Republic of China

## Abstract

The prognostic value of plasma Epstein-Barr virus (EBV) DNA remains unknown in nasopharyngeal carcinoma (NPC) treated with intensity-modulated radiation therapy (IMRT). We retrospectively reviewed medical records of 584 newly diagnosed patients with nonmetastatic and biopsy-proven NPC treated using IMRT. Plasma EBV DNA concentration was measured before therapy (pre-DNA) and within 1 month of completing therapy (post-DNA) using real-time quantitative polymerase chain reaction. Receiver operating characteristic (ROC) curves were generated to identify pre-DNA and post-DNA cut-off values. Prognostic value was assessed using a multivariate Cox proportional hazards model .Three-year disease-free survival (DFS), overall survival (OS), loco-regional relapse-free survival (LRRFS) and distant metastasis-free (DMFS) for pre-DNA >2010 vs.≤2010 were 78.1% vs. 93.6% (*P* < 0.001), 92.3% vs. 98.9% (*P* < 0.001), 90.9% vs. 96.6% (*P* = 0.004) and 85.5% vs. 96.6% (*P* < 0.001), respectively. Three-year DFS, OS, LRRFS and DMFS for post-DNA >0 vs. = 0 were 49.9% vs. 88.5% (*P* < 0.001), 72.1% vs. 97.5% (*P* < 0.001), 86.6% vs. 94.3% (*P* *=* 0.019), and 60.5% vs. 93.3% (*P* < 0.001), respectively. Plasma EBV DNA remains a prognostic factor in IMRT era and should be incorporated into TNM staging to guide individualized treatment strategies in NPC.

Nasopharyngeal carcinoma (NPC) has an obviously unbalanced geographical distribution, with the highest incidence occurring in southern China, where it ranges between 15 and 50 cases per 100,000 of the population^1^. As a result of anatomic constraints and its high degree of radiosensitivity, radiotherapy is the main treatment for NPC. The tumour-node-metastasis (TNM) staging system has traditionally been the most important prognostic factor for NPC^2^. However, the TNM staging system is only based on anatomical features and does not include other effective molecular markers that correlate with prognosis.

NPC has been proven to be an Epstein-Barr virus (EBV)-associated cancer^3^. With developments in molecular biology, the circulating tumour DNA concentration in the plasma and serum of patients with NPC can be quantified using real-time quantitative polymerase chain reaction (PCR). Recently, plasma EBV DNA has become widely used in clinical work^3^,^4^,^5^ and has been established as a reliable biomarker for detection, monitoring and prognostic prediction in NPC^6^,^7^,^8^.

In recent years, intensity-modulated radiation therapy (IMRT) has gradually replaced 2D-CRT as the primary radiotherapy technique. IMRT has an improved tumour target conformity and radiobiological efficacy, which leads to superior disease control and a lower treatment toxicity profile^9^. Nevertheless, few studies have reported the relationship between the plasma EBV DNA concentration and the prognosis of patients with NPC treated with IMRT. Additionally, there is no data on the prognostic value of the pre-treatment EBV DNA (pre-DNA) concentration and plasma EBV DNA concentration at the first evaluation after radiotherapy (within 1 month; post-DNA) in patients with NPC treated with IMRT.

On the basis of this premise, we conducted a retrospective study to explore the long-term prognostic impact of pre-DNA and post-DNA on the outcome of patients with NPC undergoing modern radiotherapy treatment.

## Materials and methods

### Study subjects

We retrospectively analysed 1811 newly diagnosed patients with nonmetastatic and biopsy-proven NPC, who were treated between November 2009 and February 2012 at Sun Yat-sen university cancer center. Patients with both pre-treatment EBV DNA and post-treatment EBV DNA were recruited for the study. The latter was obtained within 1 month of treatment, at the first evaluation after radiotherapy. In total, 584 patients were analysed. This study was approved by the Research Ethics Committee of Sun Yat-sen university cancer center. Informed consent was obtained from all the patients. The methods used in this study were in accordance with the approved guidelines.

### Clinical staging

The routine staging workup included a complete history and clinical examinations of the head and neck region, direct fibre-optic nasopharyngoscopy, magnetic resonance imaging (MRI) scans of the skull base and whole neck, chest radiography, whole-body bone scan and abdominal sonography, as well as positron emission tomography (PET)-CT (for 189 [32.4%] patients). The pre-treatment physical condition evaluations included a complete blood count, biochemical profile, coagulation test, electrocardiogram (ECG) and infectious dsease assessment (hepatitis, HIV, syphilis). Tumour-related markers were quantified, including immunoglobulin A (IgA) antibodies to EBV viral caspid antigen (VCA) and EBV early antigen (EA), and the plasma EBV DNA load. All patients received a dental evaluation before radiotherapy.

All patients were restaged according to the 7^th^ edition of the International Union against Cancer/American Joint Committee on Cancer (UICC/AJCC) system^10^. All MRI materials and clinical records were reviewed to minimize heterogeneity in restaging. Two radiologists employed at our hospital separately evaluated all of the scans and disagreements were resolved by consensus.

### DNA extraction and real-time quantitative polymerase chain reaction

Before therapy and within 1 month after the completion of radiotherapy, peripheral blood (3 ml) was collected from each patient, placed in an ethylene diamine tetra acetic (EDTA)-coated tube, and centrifuged at 1600 g for 15 min to isolate plasma and peripheral blood cells (PBC). The plasma samples were stored at −80 °C until further processing. DNA was extracted from plasma and PBC using the QIAamp Blood Kit (Qiagen, Hilden, Germany) and the “blood and body fluid protocol” recommended by the manufacturer. A total of 500 μl of each plasma sample was used for DNA extraction per column and the final elution volume was 50 μl per column.

The concentration of EBV DNA in the plasma was measured using a real-time quantitative PCR assay targeting the *BamH* I-W region of the EBV genome. The sequences of the forward and reverse primers were: 5′-GCCAG AGGTA AGTGG ACTTT-3′ and 5′-TACCA CCTCC TCTTC TTGCT-3′ respectively. A dual fluorescently-labelled oligomer, 5′-(FAM)CACAC CCAGG CACAC ACTAC ACAT(TAMRA)-3′ served as the probe. Sequence data for the EBV genome were obtained from the GenBank sequence database. The principles of the real-time quantitative PCR assay and detailed reaction setup procedures were as described previously^4^,^11^. The plasma EBV DNA concentration was calculated using the following equation: *C* *=* *Q* × (*V*_*DNA*_*/V*_*PCR*_) × *(1/V*_*EXT*_), in which *C* represents the target concentration in plasma (copies/ml), *Q* represents the target quantity (copy number) determined by PCR, *V*_*DNA*_ represents the total volume of DNA obtained after extraction (typically 50 μl/Qiagen extraction), *V*_*PCR*_ represents the volume of DNA solution used for PCR (typically 2 μl) and *VEXT* represents the volume of plasma extracted (typically 0.5 ml)^11^.

### Clinical treatment

#### Radiotherapy

All patients received IMRT at Sun Yat-sen university cancer center. Immobilization was carried out using a custom-made head-to neck-thermoplastic cast with the patient’s neck resting on a support. A high-resolution planning computed tomography scan with contrast was taken from the vertex to 2 cm below the sternoclavicular joint at a slice thickness of 3 mm. Target volumes were delineated slice-by-slice on treatment planning CT scans using an individualized delineation protocol that complies with International Commission on Radiation Units and Measurements reports 50 and 62. The prescribed doses were 66–72 Gy at 2.12–2.43 Gy/fraction to the planning target volume (PTV) of the primary gross tumour volume (GTVnx), 64–70 Gy to the PTV of the GTV of the involved lymph nodes (GTVnd), 60–63 Gy to the PTV of the high-risk clinical target volume (CTV1), and 54–56 Gy to the PTV of the low-risk clinical target volume (CTV2). All targets were treated simultaneously using the simultaneous integrated boost technique.

#### Chemotherapy

According to our institutional guidelines, prior to commencing treatment we recommended radiotherapy alone for stage I disease, concurrent chemoradiotherapy for stage II disease, and concurrent chemoradiotherapy (CCRT)+/− neoadjuvant/adjuvant chemotherapy for stage III to IVA-B disease. Of the 584 patients, 197 (33.7%) patients received both neodajuvant chemotherapy (NCT) and concurrent chemotherapy (CCRT), 36 (6.2%) patients received only NCT, 256 (43.8%) patients received only CCRT and 95 (16.3%) patients received IMRT alone. Only eight patients (1.4%) received adjuvant chemotherapy. Neoadjuvant or adjuvant chemotherapy consisted of cisplatin with 5-fluorouracil, cisplatin with taxoids or triple agent treatment with cisplatin, 5-fluorouracil and toxoids every three weeks for two or three cycles. Concurrent chemotherapy consisted of cisplatin given weekly or on weeks 1, 4 and 7 of radiotherapy.

### Follow-up and statistical analysis

Patient follow-up was measured from the first day of therapy to the day of last examination or death. Patients were examined at least every 3 months during the first 2 years, with follow-up examinations every 6 months thereafter until death. The end points (time to the first defining event) included disease-free survival (DFS), overall survival (OS), loco-regional relapse-free survival (LRRFS) and distant metastasis-free survival (DMFS).

Receiver operating characteristic (ROC) curve analysis was used to calculate the cut-off values for pre-DNA and post-DNA. The Chi-square test was used to evaluate the association of EBV DNA concentrations with tumour staging (T classification, N classification and overall stage). Life-table estimation was performed using the Kaplan-Meier method and the differences were compared using the log-rank test. The multivariate Cox proportional hazards model was used to estimate the hazard ratios (HR) and calculate 95% confidence intervals (CI). Variables in the model included age, gender, pathologic type, T classification, N classification, chemotherapy, pre-DNA and post-DNA. All statistical tests were two-sided; *P* < 0.05 was considered statistically significant. Stata Statistical Package (STATA 12; StataCorp LP, College Station, TX, USA) was used for all analysis.

## Results

Of the 584 patients, 436 (74.7%) were male and 148 (25.3%) were female; the median age was 44 years (range 14–78). The clinical characteristics of the patients before treatment are listed in Table 1. The median follow-up time was 38.2 months (range 4.6–58.6). By the last follow-up, 24 (4.1%) patients had developed local recurrence, 19 (3.3%) had developed regional recurrence, 56 (9.6%) had developed distant metastases, 7 (1.2%) had developed both local recurrence and distant metastases, and 32 (5.5%) patients had died. The 3-year DFS rate, OS rate, LRRFS rate and DMFS rate for the entire cohort were 85.3%, 95.4%, 93.6% and 90.7%, respectively.

### Pre-treatment plasma EBV DNA

In total, 454 (77.7%) patients had detectable pre-DNA. The relationship between pre-DNA and TNM staging is presented in Table 2. Pre-DNA was strongly associated with T classification (χ^2^ = 150.123, *P* < 0.001), N classification (χ^2^ = 557.592, *P* < 0.001) and overall stage (χ^2^ = 212.644, *P* < 0.001). There was no statistically significant association in pre-DNA between patients with loco-regional recurrence and patients with distant metastasis (*P* = 0.965); however, patients who suffered loco-regional recurrence (*P* = 0.008) or distant metastasis (*P* < 0.001) had significantly higher pre-DNA levels than patients who remained disease-free.

**Figure 1:**
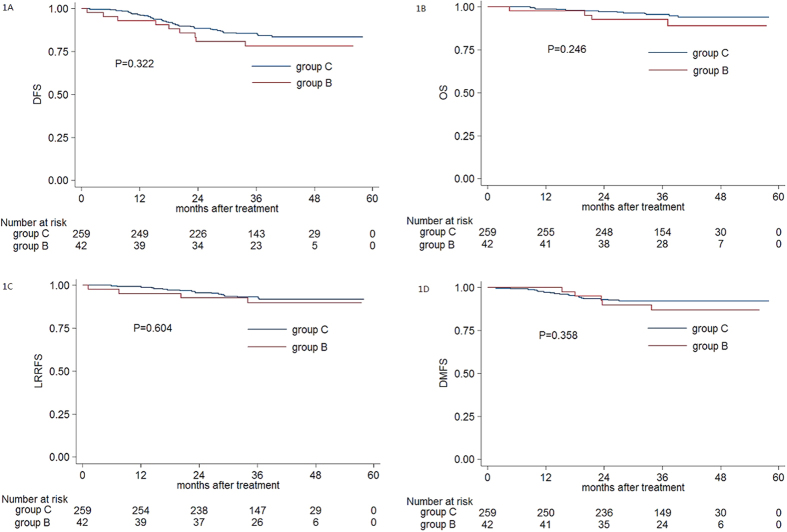
Kaplan-Meier DFS (**A**) OS (**B**) LRRFS (**C**) and DMFS (**D**) curves for patients with NPC stratified as the T_1–2_N_2–3_ and T_3–4_N_0–1_ group. Abbreviations: DFS=disease-free survival; OS=overall survival; LRRFS *=* local-regional relapse-free survival; DMFS *=* distant metastasis-free survival.

To compare the impact of local advanced stage and regional advanced stage disease on pre-treatment EBV DNA, patients were divided into four subgroups: (A) early local and regional stage (T1-2N0-1); (B) early local stage and advanced regional stage (T1-2N2-3); (C) advanced local stage and early regional stage (T3-4N0-1); (D) advanced local and regional stage (T3-4N2-3). Median pre-DNA levels were 6040 copies/ml (interquartile range, 387–21175) for the 43 patients (7.4%) in group B and 2550 copies/ml (interquartile range, 80–20200) for the 259 patients (44.3%) in group C; these values were not significantly different (*P* *=* 0.216). The 3-year DFS rate, OS rate, LRRFS rate, and DMFS rate for patients with T_1–2_N_2–3_ vs. T_3–4_N_0–1_ were 85.5% vs. 78.2% (*P* *=* 0.322, Fig. 1A), 95.6% vs. 92.7% (*P* *=* 0.246, Fig. 1B), 93.1% vs. 89.7% (*P* *=* 0.604, Fig. 1C), and 92.2% vs. 86.9% (*P* *=* 0.358, Fig. 1D), respectively.

**Figure 2:**
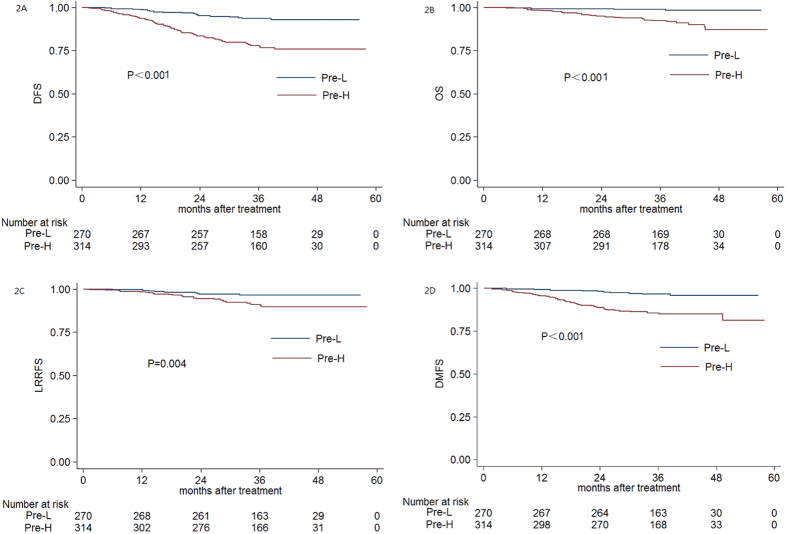
Kaplan-Meier DFS (**A**) OS (**B**) LRRFS (**C**) and DMFS (**D**) curves for patients with NPC stratified as the Pre-H and Pre-L group; Pre-H group *=* patients with a pre-treatment EBV DNA≥2010 copies/ml; Pre-L group *=* patients with a pre-treatment EBV DNA <2010 copies/ml. Abbreviations: Pre-H *=* high pre-treatment EBV DNA; Pre-L *=* low pre-treatment EBV DNA.

According to ROC curve analysis, the pre-DNA cut-off values were 2010 copies/ml for DFS (AUC, 0.636; sensitivity, 0.798; specificity, 0.509) and 2010 copies/ml for DMFS (AUC, 0.636; sensitivity, 0.821; specificity, 0.492) . The cut-off value for DFS was used in the univariate analysis. The 3-year DFS rate, OS rate, LRRFS rate and DMFS rate for patients with a pre-DNA>2010 vs.≤2010 were 78.1% vs. 93.6% (P < 0.001, Fig. 2A), 92.3% vs. 98.9% (P < 0.001, Fig. 2B), 90.9% vs. 96.6% (P = 0.004, Fig. 2C) and 85.5% vs. 96.6% (P < 0.001, Fig. 2D), respectively.

### Post-treatment plasma EBV DNA

Post-DNA was detectable in 50 (8.6%) patients, and was detected in 25 (5.1%) patients who remained disease-free, 6 (16.2%) patients who suffered locoregional recurrence and 19 (33.9%) patients who developed distant metastasis. Among the 25 (4.3%) patients who remained disease-free, post-DNA levels were undetectable within the first two weeks after radiotherapy in 7 (28%) patients, within 3 months in 10 (40%) patients and within 6 months in 8 (32%) patients. The median post-DNA concentrations for patients with distant metastasis and patients with locoregional recurrence were 0 copies/ml, and these values were not significantly different (Kruskal-Wallis rank-sum test, *P* *=* 0.129). Based on ROC curve analysis, the post-DNA cut-off value for DFS was 20 copies/ml (AUC, 0.617; sensitivity, 0.281; specificity, 0.949); however, we selected 0 copy/ml as the cut-off value.

**Figure 3:**
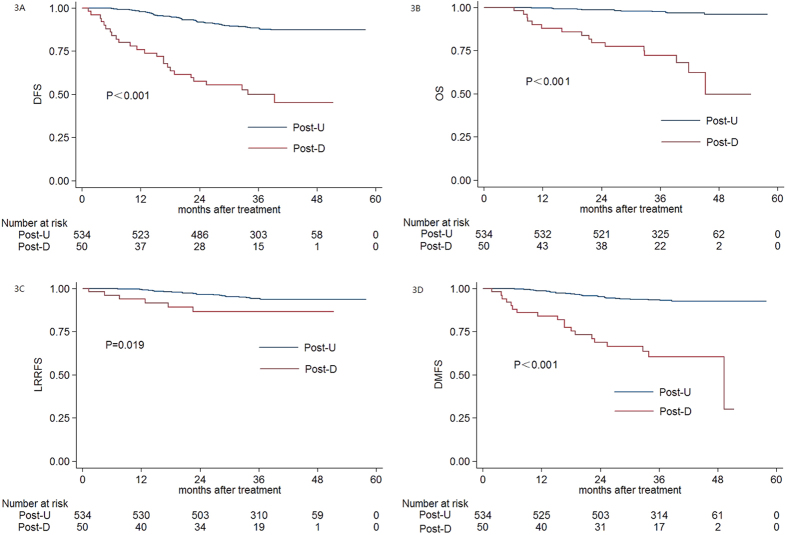
Kaplan-Meier DFS (**A**) OS (**B**) LRRFS (**C**) and DMFS (**D**) curves for patients with NPC stratified as the Post-U and Pre-D group; Post-U group *=* patients with a post-treatment EBV DNA *=* 0 copies/ml; Post-D group *=* patients with a post-treatment EBV DNA >0 copies/ml. Abbreviations: Post-U *=* undetectable post-treatment EBV DNA; Post-D *=* detectable post-treatment EBV DNA.

Univariate analysis showed that the 3-year DFS rate, OS rate, LRRFS rate, and DMFS rate for the patients with detectable post-DNA (post-D) and undetectable post-DNA (post-U) were 49.9% vs. 88.5% (*P* < 0.001, Fig. 3A), 72.1% vs. 97.5% (*P* < 0.001, Fig. 3B), 86.6% vs. 94.3% (*P* *=* 0.019, Fig. 3C), and 60.5% vs. 93.3% (*P* < 0.001, Fig. 3D), respectively. Patients with detectable post-DNA had a significantly poorer prognosis than patients with undetectable post-DNA.

### Combination of the pre-treatment and post-treatment plasma EBV DNA status

**Figure 4:**
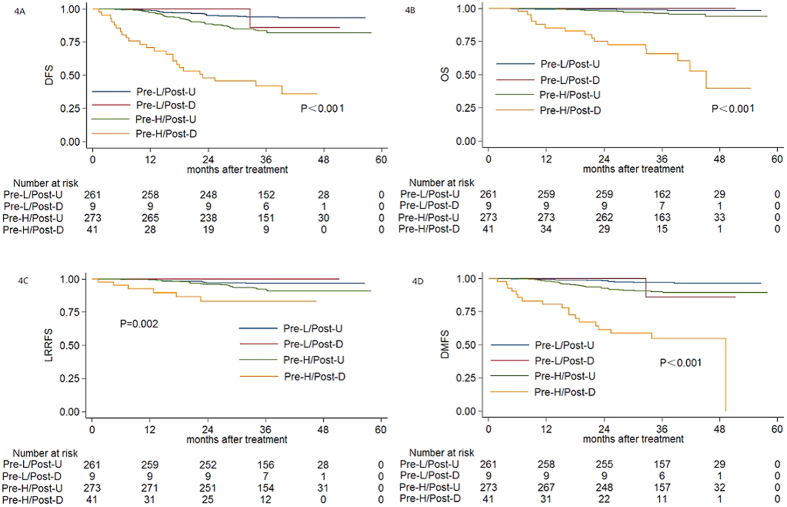
Kaplan-Meier DFS (**A**) OS (**B**) LRRFS (**C**) and DMFS (**D**) curves for patients with NPC stratified as the Pre-L/Post-U, Pre-L/Post-D, Pre-H/Post-U and Pre-H/Post-D group.

**Figure 5:**
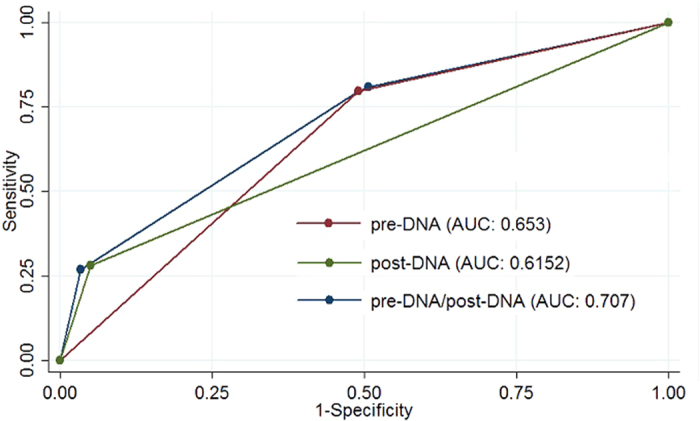
ROC curve analysis in comparing the prognostic value of pre-DNA, post-DNA and the combination of pre-DNA and post-DNA. Abbreviations: ROC *=* Receiver operating characteristic; AUC *=* area under curve.

According to the baseline plasma EBV DNA level and the change in plasma EBV DNA after radiotherapy, patients were classified into four subgroups: (1) low pre-DNA (<2010 copies/ml, Pre-L) and post-U; (2) low pre-DNA (<2010 copies/ml, Pre-L) and post-D; (3) high pre-DNA (≥2010 copies/ml, Pre-H) and post-U; (4) high pre-DNA (≥2010 copies/ml, Pre-H) and post-D. The 3-year DFS rate, OS rate, LRRFS rate and DMFS rate for the four subgroups were 93.8% vs. 85.7% vs. 83.4% vs. 41.9% (*P* < 0.001, Fig. 4A), 98.8% vs. 100% vs. 96.1% vs. 65.8% (*P* < 0.001, Fig. 4B), 96.5% vs. 100% vs. 92.0% vs. 83.2% (*P* < 0.001, Fig. 4C), and 96.9% vs. 85.7% vs. 89.9% vs. 54.7% (*P* < 0.001, Fig. 4D), respectively. In addition, the AUC for pre-DNA, post-DNA and the combination of pre-DNA and post-DNA with respect to DFS were 0.653, 0.615 and 0.707, respectively (Fig. 5).

### Cox multivariate analysis

Multivariate analysis was performed to adjust for various prognostic factors. In agreement with the results of univariate analysis, both pre-DNA and post-D were identified as unfavourable prognostic factors. Pre-DNA (*P* < 0.001) and post-DNA (*P* < 0.001) were independent prognostic factors for DFS, pre-DNA (*P* *=* 0.005) and post-DNA (*P* < 0.001) and age (*P* *=* 0.04) were independent prognostic factors for OS, pre-DNA (*P* *=* 0.002) and post-DNA (*P* < 0.001) were independent prognostic factors for DMFS, and pre-DNA (*P* *=* 0.006) was a independent prognostic factor for LRRFS (Table 3).

## Discussion

Pre-DNA and post-DNA have been reported to have prognostic value in patients with non-metastatic NPC treated with 2D or 3D radiotherapy^12^,^13^ and patients with metastatic NPC treated with IMRT^5^. The current study is the first assessment of the prognostic value of pre-DNA and post-DNA in a large cohort of patients with non-metastatic NPC treated with IMRT, and demonstrates that both pre-DNA and post-DNA are significant prognostic factors in patients with non-metastatic NPC receiving IMRT.

With the development of molecular biology, a number of biological markers have been identified as prognostic factors in NPC. The demonstration that tumour-derived genetic alternations can be detected in the plasma and serum of patients with cancer suggests that a component of circulating DNA is released by tumour cells^14^,^15^,^16^ Studies have indicated that the circulating cell-free EBV DNA is largely released from apoptotic and necrotic cancer cells. Therefore, circulating cell-free DNA could represent the tumour burden^17^.

This study demonstrates that pre-DNA is strongly associated with T classification and N classification, in agreement with previous studies^11^,^12^,^13^,^18^ which reported that pre-DNA is derived from tumour cells and correlates with the tumour volume. This relationship was further assessed. Although no statistically significant association was observed in pre-DNA and 3-year DFS, OS, LRRFS and DMFS, patients with T_1–2_N_2–3_ had poorer 3-year DFS and DMFS than patients with T_3–4_N_0–1_, and this effect became more significant after three years (Figs. 1A and 1D). Thus, pre-DNA may not precisely predict the tumour burden for patients with advanced N stage disease as the circulating cell-free plasma EBV DNA load only originates from apoptotic and necrotic tumour cells, rather than all circulating tumour cells^6^.

In this study, both pre-DNA and post-DNA were strongly associated with 3-year DFS, OS, LRRFS and DMFS, in agreement with previous reports that pre-DNA^4^,^19^,^20^ and post-DNA^5^,^19^,^21^ were important prognostic factors in patients with NPC treated with 2D or 3D conventional radiotherapy. Patients with Pre-H and post-D EBV DNA had a poorer prognosis, presumably as these patients usually have a larger tumour burden before treatment and higher risk of tumour recurrence or distant metastasis after treatment. Plasma EBV DNA was confirmed to be a reliable marker of prognosis for patients with NPC treated with IMRT in this study. However, a recently published study showed that patients with positive pre-DNA achieved superior DFS to patients with negative pre-DNA^22^; one reasonable explanation for this difference is that other clinicopathological factors that influence prognosis were not balanced between groups. The cut-off values for pre-DNA and post-DNA in this study were 2010 copies/ml and 20 copies/ml, respectively. Compared to the lower cut-off values used in previous studies^4^, a higher proportion of patients with a poor prognosis would be detected using the cut-off values identified in this study^19^,^23^,^24^. Considering the cut-off value identified in ROC curve analysis was close to 0 copy/ml and there was an extreme difference in the prognosis of patients with Post-D and Post-U, we would like to suggest that the post-DNA cut-off value should be set to 0 copy/ml for patients treated with IMRT and adjuvant chemotherapy may be helpful. Moreover, the cut-off values for pre-DNA with respect to DMFS indicate that patients with a pre-DNA concentration above 2010 copies/ml may have micro-metastasis at other sites before treatment and could benefit from neoadjuvant chemotherapy.

Furthermore, by combining the pre-DNA level and post-DNA status, we identified that the Pre-H/post-D subgroup have an extremely poor prognosis. Additionally, by comparing the ROC curves, the combination of pre-DNA and post-DNA had greater prognostic value than pre-DNA or post-DNA alone (Fig. 5). Notably, the prognosis of patients with Pre-L/Post-D was not as poor as expected, and was even better than previous reports^4^. This may be due to that fact that 77.8% (7/9) of patients in this subgroup had detectable post-D within the first two weeks after radiotherapy, which then reduced to undetectable without adjuvant therapy. Patients whose post-D rapidly reduces to undetectable may achieve a favourable prognosis and may not require adjuvant chemotherapy. However, this result could be affected by the small sample size for this subgroup. However, the pre-DNA or post-DNA between patients with metastatic disease and loco-regional recurrent disease were not significantly different, which indicates that both pre-DNA and post-DNA can only predict disease failure, not the pattern of failure.

The national comprehensive cancer network (NCCN) guidelines recommend CCRT+adjuvant chemotherapy (ACT; category 2A), CCRT (category 2B) or CCRT+neoadjuvant chemotherapy (NCT; category 3) for stage II-IV NPC patients. However, these therapeutic decisions are only based on TNM staging. Moreover, our previous studies showed that both NCT^25^ and ACT^26^ do not improve prognosis. However, we did not select patients who may benefit from NCT or ACT based on pre-DNA or post-DNA. According to the outcomes of the present study, we would like to suggest NCT+CCRT for patients with Pre-H, ACT+CCRT for patients with persistent post-D, and NCT+CCRT+ACT for patients with pre-H/post-D. However, toxicity and tolerance should be considered when ACT is indicated. ACT regimens also require further research; a number of clinical trials are currently in progress (NCT02363400, NCT00370890, NCT02143388).

This study demonstrates that plasma EBV DNA is a reliable biomarker of prognosis in patients treated with IMRT. Therefore, plasma EBV DNA should be incorporated into disease staging to address the shortcomings of the current TNM staging system and guide clinical treatment. However, this study is limited by its retrospective nature and fact the follow-up time may be insufficient, though we chose DFS as the major endpoint to address this shortcoming. Lin *et al.* also reported that the plasma EBV DNA clearance rate was a prognostic biomarker of metastasis or recurrence in patients with NPC^27^, which represents an important direction for future research. Additional clinical trials should be designed to investigate the prognostic value of the EBV DNA clearance rate in patients with non-metastatic NPC.

## Conclusions

Plasma EBV DNA remains an important prognostic factor in patients with NPC treated with IMRT. Plasma EBV DNA should be considered when selecting treatment strategies. Furthermore, plasma EBV DNA should be included in the TNM staging system to enhance its accuracy and guide individualized treatment strategies in order to improve the treatment outcomes of patients with NPC.

## Additional Information

**How to cite this article**: Peng, H. *et al.* Prognostic Impact of Plasma Epstein-Barr Virus DNA in Patients with Nasopharyngeal Carcinoma Treated using Intensity-Modulated Radiation Therapy. *Sci. Rep.* **6**, 22000; doi: 10.1038/srep22000 (2016).

## Figures and Tables

**Table 1:** Baseline characteristics of the 584 patients with NPC.

Characteristic	No. of cases (%)
Age (years)	
≥50	179 (30.7)
<50	405 (69.3)
Sex	
Male	436 (74.7)
Female	148 (25.3)
Pathology (WHO classification)	
Keratinizing	36 (6.2)
Non-keratinizing	548 (93.8)
T classification*	
T1	109 (18.7)
T2	104 (17.8)
T3	274 (46.9)
T4	97 (16.6)
N classification*	
N0	98 (16.8)
N1	332 (56.8)
N2	111 (19.0)
N3	43 (7.4)
Overall stage*	
I	34 (5.8)
II	137 (23.5)
III	281 (48.1)
IVA-B	132 (22.6)
Chemotherapy	
Yes	492 (84.2)
No	92 (15.8)
Induction chemotherapy	
Yes	233(39.9)
No	351(60.1)
Concurrent chemotherapy	
Yes	453 (77.6)
No	131 (22.4)

Abbreviations: *According to the 7^th^ edition of the AJCC/UICC staging system; WHO *=* World Health Organization; NPC *=* nasopharyngeal carcinoma.

**Table 2:** Associations between pre-DNA and TNM staging.

Characteristic	No. of cases	Median (copies/ml)	Interquartile range	*P*-value^a^
T classification^b^				<0.001
T1	109	660	0–4380	
T2	104	2100	80–14400	
T3	274	3200	120–16750	
T4	97	6750	1450–68700	
N classification^b^				<0.001
N0	98	360	0–4450	
N1	332	2170	70–15800	
N2	111	6240	630–28600	
N3	43	12400	4320–72400	
Overall stage^b^				<0.001
I	34	0	0–1640	
II	137	1200	0–6300	
III	281	2650	80–13600	
IV	132	8160	2050–58950	
Disease failure				0.965
Loco–regional	43	2440	60–14700	
Distant	56	2130	40–13830	

Abbreviations: ^a^P-values were calculated using the Chi-square test or Fisher’s exact test, as indicated.

^b^According to the 7^th^ edition of the AJCC/UICC staging system.

**Table 3:** Multivariate analysis of prognostic factors in patients with NPC.

Endpoint	Characteristic	HR	95% CI	*P-*value^a^
OS	Pre-DNA	4.581	1.583–13.257	0.005
	Post-DNA	11.314	5.552–23.057	<0.0001
	Age	2.125	1.037–4.355	0.04
DMFS	Pre-DNA	3.046	1.514–6.127	0.002
	Post-DNA	5.632	3.204–9.899	<0.0001
LRRFS	Pre-DNA	2.889	1.363–6.123	0.006
DFS	Pre-DNA	3.190	1.890–5.384	<0.0001
	Post-DNA	4.908	3.069–7.849	<0.0001

Abbreviations: OS *=* overall survival; DMFS *=* distant metastases-free survival; LRRFS *=* loco-regional relapse-free survival; DFS *=* disease-free survival; HR *=* hazard ratio; CI *=* confidence interval; pre-DNA *=* pre-treatment EBV DNA; post-DNA *=* post-treatment EBV DNA.

a: Multivariate *P*-values were calculated using an adjusted Cox proportional-hazards model. The following parameters were included in the Cox proportional hazards model with backward elimination: age (≥50 y vs. <50 y), gender (male vs. female), T classification (T_3–4_ vs. T_1–2_), N classification (N_2–3_ vs. N_0–1_), use of chemotherapy (yes vs. No), pathological type (keratinizing vs. non-keratinizing), pre-DNA (≥2010 copies/ml vs.<2010 copies/ml), post-DNA (>0 opy/ml vs.0 copy/ml).
